# Multilevel Analysis of Air Pollution and Early Childhood Neurobehavioral Development

**DOI:** 10.3390/ijerph110706827

**Published:** 2014-07-02

**Authors:** Ching-Chun Lin, Shih-Kuan Yang, Kuan-Chia Lin, Wen-Chao Ho, Wu-Shiun Hsieh, Bih-Ching Shu, Pau-Chung Chen

**Affiliations:** 1 Institute of Occupational Medicine and Industrial Hygiene, National Taiwan University College of Public Health, 17 Syujhou Road, Taipei 10055, Taiwan; E-Mails: ccleuven@gmail.com (C.-C.L.); skyang530@gmail.com (S.-K.Y.); 2 Department of Nursing, National Taipei University of Nursing and Health Sciences, Taipei 10845, Taiwan; E-Mail: kuanchia@ntunhs.edu.tw; 3 Institute of Environmental Medicine, China Medical University, Taichung 40402, Taiwan; E-Mail: wcho@mail.cmu.edu.tw; 4 Department of Pediatrics, National Taiwan University College of Medicine and Hospital, Taipei 10055, Taiwan; E-Mail: hsiehws@ntu.edu.tw; 5 Institute of Allied Health Sciences and School of Nursing, National Cheng Kung University, Tainan 701, Taiwan; E-Mail: shubih@mail.ncku.edu.tw; 6 Department of Public Health, National Taiwan University College of Public Health, Taipei 10055, Taiwan; 7 Department of Environmental and Occupational Medicine, National Taiwan University College of Medicine and Hospital, Taipei 10055, Taiwan

**Keywords:** air pollution, neurobehavioral development, trimester, multilevel analysis

## Abstract

To investigate the association between the ambient air pollution levels during the prenatal and postnatal stages and early childhood neurobehavioral development, our study recruited 533 mother-infant pairs from 11 towns in Taiwan. All study subjects were asked to complete childhood neurobehavioral development scales and questionnaires at 6 and 18 months. Air pollution, including particulate matter ≤10 μm (PM_10_), carbon monoxide (CO), sulfur dioxide (SO_2_), nitrogen dioxide (NO_2_), ozone (O_3_), and hydrocarbons, was measured at air quality monitoring stations in the towns where the subjects lived. Multilevel analyses were applied to assess the association between air pollution and childhood neurobehavioral development during pregnancy and when the children were 0 to 6 months, 7 to 12 months, and 13 to 18 months old. At 18 months, poor subclinical neurodevelopment in early childhood is associated with the average SO_2_ exposure of prenatal, during all trimesters of pregnancy and at postnatal ages up to 12 months (first trimester β = −0.083, se = 0.030; second and third trimester β = −0.114, se = 0.045; from birth to 12 months of age β = −0.091, se = 0.034). Furthermore, adverse gross motor below average scores at six months of age were associated with increased average non-methane hydrocarbon, (NMHC) levels during the second and third trimesters (β = −8.742, se = 3.512). Low-level SO_2_ exposure prenatally and up to twelve months postnatal could cause adverse neurobehavioral effects at 18 months of age. Maternal NMHC exposure during the 2nd and 3rd trimesters of pregnancy would be also associated with poor gross motor development in their children at 6 months of age.

## 1. Introduction

Previous studies found that outdoor air pollution has neurotoxic and behavioral effects on humans or animals. Carbon monoxide (CO) interferes with oxygen transport through the formation of carboxyhemoglobin and exposure to carboxyhemoglobin levels of 2.1% to 4.2%. Carboxyhemoglobin resulted in a significant decrease in video game performance and may impair humans’ higher cognitive functions [1]. Even low-level exposure to CO impairs higher cognitive functions [2]. In animal studies, prenatal exposure to nitrogen dioxide (NO_2_) affected neuromuscular coordination and caused functional deficits in the developing offspring of mice [3] and prenatal sulfur dioxide (SO_2_) exposure reduced the levels of several mouse social/agonistic behaviors, such as tail rattling, freezing, and defensive postures [4] impaired neuromuscular coordination [5] and impaired the coordination and locomotor development of their offspring [6]. Exposure to high ozone (O_3_) concentrations during gestation induces permanent cerebellar damage in rats and negatively impacts activity/exploration and social behavior [7,8]. It is also found the significant increase in freezing and defensive postures, decreases in nose-sniffing behavior and decreased nerve growth factor (NGF) levels in the hippocampus [9].

In humans evidence shows that some of the pollutants can affect the developing brain and adversely affect cognitive development [10] PAH pollutants can exert genotoxic effects which inducing mutations as epigenetic effects [11]. Molecular epidemiologic studies also found that the fetus is more susceptible than adults to the effects of PAH and other toxicants [12].

The PAH–DNA adducts in cord blood were associated with delay of developmental [10,13,14], IQ [11], child behavior symptom scores of Anxious/Depressed, and Attention Problems [15]. In addition, higher levels of cord PAH–DNA adducts have been associated with reduced scores on neurocognitive tests, alone or in combination with environmental tobacco smoke (ETS) [10,11,15,16,17].

There is limited evidence on the relationship between air pollutant exposure and early childhood neurodevelopment, and the neurological effects of air pollution remain unclear [18]. The developing nervous system is an extremely sensitive target, and the blood-brain barrier is not fully developed until the middle of the first year of life [19]. Our study aimed to investigate the relationship between exposure to ambient air pollutants during the prenatal and postnatal periods and with early childhood neurodevelopment.

## 2. Material and Methods

### 2.1. Study Population

A prospective cohort study was conducted between October 2003 and January 2004 as pilot survey of Taiwan Birth Cohort Pilot Study (TBCS-p). The participants of TBCS-p were randomly selected from babies born between October 2003 and January 2004 in 29 villages or cities in Taiwan and were followed up when they were 6, 18 months old. There are 629 mother-infant pairs agreed to participate in the study. There were 613 mother-infant pairs from 11 towns with air quality monitoring stations operated by the Taiwan Air Quality Monitoring Network (TAQMN) [20]. We excluded infants whose mothers smoked during the gestational period (*N* = 10) and infants with congenital defects (*N* = 22), preterm birth (*N* = 31), or low birth weight (*N* = 33). A total of 533 mother-infant pairs were recruited for this study. All study participants provided prior informed consent, as required by the Ethics Review Board of the National Taiwan University College of Public Health.

### 2.2. Exposure Assessment

The TAQMN was established in 1993 by the Environmental Protection Administration. Ambient levels of key air pollutants are measured and reported on an hourly basis. All instruments in TAQMN stations are maintained and periodically calibrated according to the TAQMN quality management plan. TAQMN monitoring stations provide hourly ambient concentrations of particulate matter ≤10 μm (PM_10_), CO, O_3_, SO_2_, NO_2_, and total hydrocarbons (THCs) and nonmethane hydrocarbons (NMHCs). In our study, hourly data were collected from the 11 air-quality monitoring stations in the towns where the study subjects inhabited.

At those monitoring stations, CO was measured by nondispersive infrared photometry (RFCA-0981-054, Thermo Environmental Instruments, Franklin, MA, USA); O_3_ was measured by ultraviolet photometric analysis (TNMH 642, Thermo Environmental Instruments); PM_10_ was measured by beta-ray absorption; SO_2_ was measured by ultraviolet fluorescence; NO_2_ was measured using a chemiluminescence analyzer (Thermo Environmental Instruments Model 42C or ECOTECH-ML 9841A, detection limit 0.4 ppb); and THCs and NMHCs were measured using a flame-ionization detector (FID; DANI Instruments-TNMH 462, Franklin, MA, USA).

The air pollutant exposure data for each child were linked from the air-quality monitoring stations of town to the exposure period from the beginning of the gestational period to eighteen months after birth. To observe the relationship between timing and magnitude of air pollutant exposure, the gestational period was divided into three trimesters of three calendar months from the mothers’ last menstrual period (LMP) to their children’s birthdays, and the period after delivery was also divided into three stages: birth to 6 months, 7 to 12 months, and 13 to 18 months for each study subject.

The traffic peaks are at around the 7 a.m and the 7 p.m. Normally, baby children would stay at home or the nearby day care center, and the outdoor activity of the children are during the daytime. We considered the daytime (7 a.m. to 7 p.m.) average levels of CO, O_3_, PM_10_, SO_2_, NO_2_, THC, and NMHC among different stages as our subjects’ exposure levels. If more than 25% of the daily average air pollutant data was missing among different stages, we did not calculate average air pollutant levels because these data might not reliably represent exposure levels during that period.

### 2.3. Data Collection

Subjects’ demographic data were obtained from the Taiwan national birth registry and the interview questionnaire. Birth certificate forms contain the following information: mother’s residential county and town, date of registration, parental identification number, birthday, education level, and marital status, and the infant’s neonatal identification number, date of birth, birth weight, gestational age, parity, singleton or multiple birth, place of delivery (hospital or clinic), and the midwife’s or obstetrician’s information. We used the infants’ dates of birth and gestational ages recorded on the birth certificates to estimate the mothers’ last menstrual period (LMP) and their children’s exposure periods. Information about maternal age, maternal education level, paternal education level, nursery type (which by parents, grandparents or others), and exposure to environmental tobacco smoke were also collected by trained interviewers using structured questionnaires, and response by the parents or the main care person at the children’s age of 6 and 18 months separately.

### 2.4. Neurobehavioral Development

The Birth Cohort Study (TBCS) was to develop an efficient developmental screening instrument for interviews in clinical and community settings. The purpose of a developmental screening tool is to survey the development of a child. It is a preventive approach to detect the problem before the child becomes deviant, so that every child can develop to his or her fullest potential the TBCS scale can be easily completed by the majority of parents. The TBCS can be used as the first stage of a two-stage developmental screening process.

The TBCS scale is a parent-reported measure of a child’s neurodevelopmental performance that consists of four developmental dimensions: gross motor, fine motor, language/communication (language), and social/self-care abilities (social). Parents were given two neurobehavioral development scales to complete at each interview. The scales contain 38 and 17 items each. Response choices consist of three ordinal categories relating to frequency (never, sometimes, and all the time). A response of “never” received one point, and “all the time” received three points. We added the scores in each dimension for each child. Both the 6- and 18-month scales have good predictive validity, and these dimensions correlate with the second edition of the Bayley Scales of Infant Development (BSID-II) [21,22].

### 2.5. Statistical Analysis

The hierarchical linear models (HLM) can be used to analyze the relationships between community level environmental data, individual risk factors, and birth outcomes. HLM is often used to study the relationship between community level factors and individual outcomes in health outcomes. Because our data were hierarchically organized and all air pollutant data were nested within different geographic areas, the associations between air pollutant exposure levels and childhood neurodevelopment were assessed via multilevel modeling analysis [23].

The analyses included individual (level one) and environmental conditions of area levels (level two). The individual level were personal variable include maternal education, maternal nationality, environmental tobacco smoke exposure, infant gestational age, breastfeeding, and parental nursery type. The area level was area air pollutant data. Using the combined level-1 and level-2 data sets, HLM provides a way to analyze the degree to which the effects of individual level variables in birth data vary systematically or randomly as a function of level-2 variables [24].

We evaluated the association between air pollutant levels during each period and neurodevelopment at 6 and 18 months of age. All analyses included individual and town exposure levels. The air pollutant and neurodevelopment data were continuous variables. The covariates for each model included maternal education (college or beyond, senior high, junior high or elementary), maternal nationality (Taiwanese, non-Taiwanese), environmental tobacco smoke exposure (never, ever), infant gestational age (weeks), breastfeeding (less than 6 months, 6 months or more), and parental nursery type. These statistical analyses were performed using SPSS for Windows, Version 11.0 (SPSS Inc., Chicago, IL, USA).

## 3. Results

The characteristics for 533 singleton live births are presented in Table 1. Most mothers in this study had high education levels, including 242 who stated they were college graduates (45.4%) and 219 senior high school graduates (41.1%), and 14.1% of mothers recruited for this study were foreign. The mean gestational age was 38.8 weeks, the mean birth weight was 3190.7 g, and about half (56.5%) of the infants were male. Among the study infants, 49.3% had prenatal environmental tobacco exposure and only 17.4% were breastfed for more than six months.

We found that gross motor scores at 6 months of age were negatively associated with the NMHC levels during the second and third trimesters. In addition, fine motor scores at 18 months of age were negatively associated with the average SO_2_ exposure levels during pregnancy and in children from birth to 12 months of age.

Table 2 shows the average air pollutant level profiles for each period. Except for ozone, all other air pollutants had an obvious peak average level between the infants’ births and six months of age. Conversely, air pollutants levels were low during the second trimester and the six to twelve month period. Most pollutants exhibit clear seasonal variations, with higher concentrations in the fall and winter.

The multilevel analysis of the relationship between air pollutant exposure and neurobehavioral development scores for children at six months and eighteen months of age are shown in Table 3 and Table 4. At six months, the increased NMHC exposure during the second and third trimesters in utero was associated with adverse gross motor scores (β = −8.742, se = 3.512). No other pollutants had a significant effect on neurobehavioral development performance at six months.

**Table 1:** Characteristic of the study subjects.

Characteristics	Study Subjects
Total	533
Maternal education, N (%)	
College +	242 (45.4)
Senior high	219 (41.1)
Junior high	52 (9.8)
Elementary	19 (3.6)
Maternal nationality, N (%)	
Taiwanese	458 (85.9)
Foreign	75 (14.1)
Gestational age, mean (SD)	38.8 (1.2)
Breast feeding ^a^, N (%)	
≥6 months	93 (17.4)
0–5 months	440 (82.6)
Environmental Tobacco Smoke (%)	
Yes	263 (49.3)
No	270 (50.7)
Gender, N (%)	
Male	301 (56.5)
Female	232 (43.5)
Birth weight, mean (SD)	3190.7 (385.5)
Parity, N (%)	
1	283 (53.1)
2	199 (37.3)
3	45 (8.4)
4	4 (0.8)
5	2 (0.4)

^a ^breast feeding was defined as including mixed and exclusive breast feeding.

At eighteen months, the increased SO_2_ exposure among the three trimesters of pregnancy and the period between birth and twelve months of age were significantly associated with a decrease in fine motor development performance scores at 18 months (first trimester β = −0.083, se = 0.030; second and third trimester β = −0.114, se = 0.045; birth to twelve months β = −0.091, se = 0.034). SO_2_ exposure from thirteen months to eighteen months after birth was not significantly associated with fine motor scores. Aside from SO_2_, all other air pollutants in this study were not significantly associated with neurobehavioral developmental scores at 18 months of age. The SO_2_ exposure during all trimesters of pregnancy and the effect from birth to 12 months on fine motor development scores at eighteen months. The prediction line shows an adverse association between increasing SO_2_ exposure levels and fine motor scores.

**Table 2:** Mean and standard deviations of air pollutant concentrations at different periods.

Air Pollutants	First Trimester			Second Trimester			Third Trimester		
	Range	Mean	SD	Range	Mean	SD	Range	Mean	SD
CO (ppm)	0.33–0.93	0.63	0.14	0.24–0.71	0.48	0.11	0.36–0.90	0.58	0.13
O_3_ (ppb)	29.86–52.52	38.87	5.30	19.07–49.18	34.32	6.35	22.65–62.84	37.45	10.18
PM_10_ (g/m^3^)	31.22–36.88	59.26	13.51	23.61–54.47	39.41	6.63	31.21–107.52	55.88	19.58
SO_2_ (ppb)	0.27–14.63	4.45	2.50	0.28–6.86	3.63	1.36	0.25–13.31	4.08	2.51
NO_2_ (ppb)	6.41–28.33	18.17	5.62	6.16–23.60	12.57	4.18	7.70–32.25	18.33	4.93
THC (ppm)	2.22–3.05	2.52	0.22	2.03–2.78	2.37	0.18	2.31–3.19	2.59	0.17
NMHC (ppm)	0.19–0.58	0.38	0.10	0.15–0.68	0.33	0.14	0.16–0.98	0.35	0.14
**Air Pollutants**	**1–6 Months**			**7–12 Months**			**13–18 Months**		
	**Range**	**Mean**	**SD**	**Range**	**Mean**	**SD**	**Range**	**Mean**	**SD**
CO (ppm)	0.37–0.93	0.66	0.14	0.21–0.71	0.45	0.11	0.40–1.01	0.67	0.14
O_3_ (ppb)	27.95–48.91	36.64	5.46	22.38–49.06	35.85	6.90	26.34–41.25	31.51	3.74
PM_10_ (μg/m^3^)	36.37–116.58	72.39	19.80	38.96–71.52	54.95	8.68	37.66–111.78	69.79	17.45
SO_2_ (ppb)	0.47–12.72	5.21	2.67	1.09–9.26	4.66	2.01	2.54–15.26	6.31	3.18
NO_2_ (ppb)	9.15–32.13	23.10	6.25	6.76–24.56	15.98	5.44	9.29–32.00	21.12	6.47
THC (ppm)	2.46–2.92	2.70	0.15	1.99–2.85	2.32	0.30	1.97–2.56	2.20	0.20
NMHC (ppm)	0.20–0.63	0.42	0.13	0.13–0.57	0.31	0.17	0.15–0.49	0.28	0.11

The average wind speed during the study period (from 2003 to 2005) was 2.00 m/s (range: 4.36 m/s to 0.68 m/s). The average ambient temperature during the study period was 23.77 °C (range: 31.44 °C to 12.51 °C). The average relative humidity during the study period was 72.13% (range: 84.38% to 32.03%).

**Table 3:** Multilevel analysis of single air pollutants and neurodevelopment scores at six months of age.

Air Pollutant in the Different Periods		Gross Motor		Fine Motor		Language		Social-Personal		Total	
		β	SE	β	SE	β	SE	β	SE	β	SE
CO	1st trimester	1.414	1.350	−0.088	0.736	−0.166	0.860	0.338	0.932	1.655	2.764
	2nd & 3rd trimester	0.459	1.695	−0.243	0.928	−0.655	1.088	−0.180	1.223	−0.528	3.477
	Birth–6 months	0.100	1.324	−0.219	0.718	−0.456	0.834	0.411	0.913	−0.043	2.697
O_3_	1st trimester	−0.056	0.044	−0.037	0.024	−0.019	0.030	0.016	0.025	−0.116	0.091
	2nd & 3rd trimester	0.001	0.028	0.003	0.016	0.012	0.020	0.009	0.022	0.030	0.058
	Birth–6 months	0.078	0.052	−0.042	0.026	−0.011	0.032	0.007	0.036	0.058	0.082
PM_10_	1st trimester	0.008	0.020	−0.007	0.011	0.003	0.011	0.017	0.016	0.033	0.044
	2nd & 3rd trimester	0.005	0.024	−0.023	0.015	−0.006	0.018	−0.007	0.021	−0.022	0.050
	Birth–6 months	−0.004	0.011	−0.006	0.007	0.003	0.008	0.001	0.009	0.006	0.022
SO_2_	1st trimester	0.015	0.072	−0.002	0.042	0.027	0.050	0.010	0.056	0.090	0.147
	2nd & 3rd trimester	−0.125	0.113	−0.037	0.062	0.036	0.073	−0.009	0.082	−0.092	0.229
	Birth–6 months	−0.053	0.070	−0.001	0.039	0.033	0.046	0.002	0.051	0.019	0.141
NO_2_	1st trimester	−0.005	0.034	−0.004	0.019	0.017	0.022	0.022	0.025	0.023	0.070
	2nd & 3rd trimester	−0.067	0.046	−0.028	0.025	−0.011	0.029	0.002	0.033	−0.110	0.093
	Birth–6 months	−0.002	0.031	−0.010	0.016	0.002	0.019	0.012	0.021	0.002	0.062
THC	1st trimester	0.123	1.233	−0.504	0.656	0.583	0.782	0.192	0.871	0.438	2.624
	2nd & 3rd trimester	−2.143	1.888	0.940	0.979	0.269	1.203	−0.970	1.335	−1.606	4.031
	Birth–6 months	−0.173	1.908	−1.451	0.996	0.523	1.210	0.087	1.346	−0.753	4.058
NMHC	1st trimester	−6.134	4.392	−2.014	2.471	3.121	2.957	−0.673	3.284	−5.721	9.843
	2nd & 3rd trimester	−8.742 *	3.512	−8.742	3.512	2.091	1.985	2.091	1.985	−3.986	8.024
	Birth–6 months	−2.822	2.696	−2.166	1.494	1.453	1.809	−0.337	2.008	−4.603	5.952

Adjusted for maternal education level, maternal nationality, gestational age, infant sex, breastfeeding, environmental tobacco smoke exposure, and nursery type (which include parents, grandparents or others). Subject: area; * Significant difference (*p* < 0.05).

**Table 4:** Multilevel analysis of single air pollutants and neurodevelopment scores at eighteen months of age.

Air Pollutant in the Different Periods		Gross Motor		Fine Motor		Language		Social-Personal		Total	
		β	SE	β	SE	β	SE	β	SE	β	SE
CO	1st trimester	0.265	0.480	0.005	0.585	1.129	0.758	0.226	0.644	1.711	1.705
	2nd & 3rd trimester	−0.059	0.603	−1.095	0.726	0.177	0.947	−0.869	0.799	−2.119	2.084
	Birth–12 months	0.297	0.542	−0.236	0.662	0.806	0.859	−0.053	0.729	0.856	1.928
	13–18 months	0.077	0.552	−0.114	0.652	0.285	0.826	−1.394	0.626	−0.650	1.832
O_3_	1st trimester	−0.012	0.017	−0.022	0.019	−0.018	0.024	0.030	0.017	−0.026	0.052
	2nd & 3rd trimester	−0.001	0.011	−0.015	0.013	0.018	0.017	0.022	0.015	0.029	0.036
	Birth–12 months	−0.013	0.023	0.014	0.025	0.044	0.031	0.046	0.032	0.112	0.084
	13–18 months	−0.014	0.024	0.011	0.028	0.017	0.035	0.033	0.033	0.053	0.075
PM_10_	1st trimester	−0.004	0.008	−0.007	0.008	0.005	0.011	0.018	0.010	0.010	0.026
	2nd & 3rd trimester	−0.009	0.010	−0.015	0.012	0.004	0.012	0.012	0.014	0.005	0.029
	Birth–12 months	−0.005	0.006	−0.011	0.007	0.004	0.009	0.006	0.009	0.001	0.020
	13–18 months	−0.004	0.005	−0.010	0.005	0.002	0.006	0.003	0.006	−0.003	0.015
SO_2_	1st trimester	−0.011	0.028	−0.083 *	0.030	0.019	0.043	0.036	0.037	−0.026	0.093
	2nd & 3rd trimester	−0.040	0.040	−0.114 *	0.045	−0.044	0.062	0.029	0.054	−0.140	0.137
	Birth–12 months	−0.017	0.031	−0.091 *	0.034	−0.010	0.047	0.009	0.041	−0.102	0.101
	13–18 months	−0.009	0.024	−0.057	0.029	−0.021	0.034	0.017	0.031	−0.073	0.075
NO_2_	1st trimester	0.003	0.013	−0.007	0.015	0.018	0.020	0.001	0.017	0.018	0.044
	2nd & 3rd trimester	−0.010	0.016	−0.029	0.020	0.003	0.026	−0.013	0.022	−0.045	0.058
	Birth–12 months	−0.003	0.013	−0.017	0.015	0.009	0.020	−0.004	0.017	−0.013	0.044
	13–18 months	−0.004	0.011	−0.016	0.013	0.004	0.018	−0.011	0.015	−0.025	0.039
THC	1st trimester	−0.229	0.387	0.478	0.529	0.287	0.700	−0.052	0.574	0.538	1.486
	2nd & 3rd trimester	0.779	0.570	−0.014	0.785	1.745	1.028	−1.524	0.842	1.071	2.202
	Birth–12 months	−0.321	0.548	0.423	0.750	0.282	0.971	−0.391	0.813	−0.118	2.107
	13–18 months	−0.343	0.647	0.094	0.887	0.383	1.172	−1.064	0.958	−0.809	2.491
NMHC	1st trimester	−0.564	1.327	−0.791	1.898	2.313	2.329	0.062	2.116	1.259	5.062
	2nd & 3rd trimester	−0.585	1.064	−1.406	1.518	0.830	1.875	0.024	1.697	−0.956	4.061
	Birth–12 months	−0.199	0.897	−0.307	1.284	1.821	1.572	0.271	1.430	1.754	3.418
	13–18 months	−0.122	1.076	−0.251	1.538	2.400	1.881	−0.211	1.717	2.168	4.104

Adjusted for maternal education level, maternal nationality, infant sex, breastfeeding, environmental tobacco smoke exposure, and nursery type. Subject: area * Significant difference (*p* < 0.05).

## 4. Discussion

Gross motor scores at 6 months of age were negatively associated with the average NMHC levels during the second and third trimesters. In addition, fine motor scores at 18 months of age were negatively associated with the average SO_2_ exposure during pregnancy and from birth to 12 months of age.

Air pollution can cause cognitive deficits and brain abnormalities, brain damage and neurogeneration [25,26,27], which has been proven in animal studies [28]. Many studies of hydrocarbons effects did not include methane. Most examined motor vehicle emissions of incompletely combusted hydrocarbons and evaporative fuel losses, which may include known neurotoxic chemicals such as toluene, xylene, ethylene, benzene, *n*-hexane, dimethylpentane, cyclohexane, and stylene. SO_2_ emissions come from the combustion of fossil fuels, such as diesel fuel, high-sulfur gasoline, and coal. Limited evidence in animal studies alone supports the effect of SO_2_ on behavioral change. Average SO_2_ concentrations were relatively low in this study. It is possible that SO_2_ is a surrogate measure of industrial emissions, and lead is one of the most dangerous neurotoxins found in air pollution. Leaded gasoline has not been used in Taiwan since 2000 and cannot be sold commercially. One study of core blood lead levels in Taiwan found that lead levels were extremely low [29].

There are some specific in our study. First, the air pollution monitoring data were collected by TAQMN and the cohort data were collected from the November and December 2003, and the air pollutant exposure data for each child were linked to the most close to the air-quality monitoring stations and the exposure period from the beginning of the gestational period to eighteen months after birth. The highly relevant of the cohort age and the exposure period could avoid confounder of the season variance were highly accurate for estimating the exposure concentrations at our study subjects’ different stages. Second, the birth registration data routinely collected by the government was also highly reliable, providing individual information on mothers and infants that could be important confounding factors.

The subjects’ estimated air pollution exposure levels at different stages were based on the average measurements made by the air quality monitoring stations in the subjects’ towns of residency. Exposure was assessed at contextual level (*i.e.*, eleven town observations) weighted by timing of the pregnancy resulting could possible minimizes or misclassification the exposure individual variations, the pollutants varying with distance from a traffic road such as CO, hydrocarbons or NO_2_. But according to the dose-response and the habit of the children care, the result should be underestimating.

Exposure misclassification might come from using outdoor air pollution to represent the exposures for infants who stayed indoors nearly all the time. In one study of indoor and outdoor air pollutants in Taiwan [30] reported that indoor concentrations of air pollutants were highly correlative with outdoor concentrations, while indoor and outdoor climate parameters were less correlated. In addition, one of the most important sources of indoor air pollutants was environmental tobacco smoke (ETS). Previous studies have indicated that prenatal exposure to ETS may affect childhood neurobehavioral development [31]. In our study, we controlled ETS exposure for indoor air pollutants. If any other indoor air pollution existed at the time, it may not have influenced our measurements and could result in an underestimation of the exposure-outcome association.

Most studies that investigated neurobehavioral development in early childhood used the Bayley Scales of Infant Development Mental Development Index [32,33] in this study, we adopted a new parental-reported scale to estimate neurobehavioral development in early childhood. The total scores from this scale were used to represent the neurobehavioral development of the studied children. We used the second edition of the Bayley Scale of Infant Development (BSID-II) as the golden standard to test the validity and consistency of the TBCS scale [34,35]. The validity of these scales includes four criteria: a developmental norm, predictive validity, validity and the ability to identify children at relative risk. In our study, the TBCS scale was used to evaluate the neurobehavioral development among these children was relatively advanced or delayed. The self-reported scales may be self-completed differentially by parental wellness may lead to non-differential misclassification. We adjusted the parental education to eliminate the confounder. However, there is still a possible chance to get type I error due to multiple testing. It warrants further research.

This study observes that low-level SO_2_ exposure prenatally and up to 12 months after birth may impair fine motor development. It is known that SO_2_ is one of the neurotoxic substances in air pollution. Previous studies about the absorption, distribution, and retention of radioactive SO_2_ in the mammalian body indicate that radioactive sulfur could be absorbed into the bloodstream of the bronchial tubes and become widely distributed to other body tissues, including the central nervous system [34,35] When SO_2_ reaches the central nervous system, its biochemical effects change the enzymatic activities of the CNS, including the activities of cofactors (NAD, FAD, and FMN) and nucleic acids (RNA, DNA, and associated proteins) [36] Moreover, SO_2_ depresses several enzymatic activities of glucose metabolism [37] These enzymes may be associated with neurobehavioral development.

The biological mechanism through which prenatal and postnatal exposure of SO_2_ could cause adverse childhood neurobehavioral development remains to be explained. However, there are several reports of SO_2_’s adverse effects on neurobehavioral development in animal studies. One study indicates that SO_2_ inhalation by mice and rabbits may increase the incidence of minor skeletal variations among their offspring [38]. In addition, maternal exposure to SO_2_ could affect neuromuscular coordination and cause functional deficits in the developing offspring. Furthermore, prenatal exposure to SO_2_ could reduce the levels of several mouse social/agonistic behaviors, such as tail rattling, freezing, and defensive postures. Fine motor performance depends on central nervous system development and neuromuscular coordination. These animal experimental studies provide us with considerable evidence.

In addition, our data about neurobehavioral development at six months of age shows that increased prenatal NMHC exposure during the second and third trimesters in utero were associated with lower gross motor development scores. NMHC is a general term for hydrocarbons other than methane. Many NMHCs are neurotoxic, such as aryl hydrocarbons [39] polycyclic aromatic hydrocarbons [40] and thiophene [41] NMHC’s adverse effects on gross motor development at six months of age may be caused by one of the NMHC components or the combined effects of two or more NMHC substances. However, NMHC monitoring data in this study was obtained from only the study areas. To verify the effect of NMHCs on childhood neurobehavioral development, further studies are needed.

There are three principles of early childhood motor development: the cephalocaudal principle, the proximodistal principle, and the hierarchical integration principle. Gross motor skills begin to develop earlier than fine motor skills, and gross skills provide the foundation for fine motor development. We observed that fine motor skills among six-month-old children were minimally variable. This may explain why SO_2_ exposure does not appear to affect fine motor development until eighteen months of age. In addition, gross motor development difference may be more significant at six months of age than at eighteen months. Although we found a no significant adverse effect of NMHC on gross motor skills at eighteen months of age, the adverse effect of NMHC was more significant at six months.

There are some limitations to this study. First, relying on air monitoring data inadequately captures intratown variability. Exposures may vary with, and more than one substance could have an effect over time. Second, development neurotoxicity might be not recognized for years or even decades. Individual vulnerability may vary, and the neurodevelopment scale may not be sufficiently sensitive to detect the effects of low-level exposure. Third, socioeconomic status may be a confounder, as it may relate to the quality and quantity of prenatal and postnatal care a child receives and influence childhood neurobehavioral development [12,42,43]. In our analysis, we adjusted for maternal education level, maternal nationality, gestational age, infant sex, breastfeeding, environmental tobacco smoke exposure, and nursery type, as a surrogate measure of the children’s home environment and socioeconomic status. Fourth, our study focuses on low levels and long-term exposure effects of air pollutants. This contrasts with previous epidemiological studies, which examined associations between air pollutants and neurobehavioral performance only in experimental acute or short-term exposure [44].

## 5. Conclusions

This study provides evidence that ambient air pollution, even low-level SO_2_ exposure, during pregnancy and up to 12 months of age is associated with poor subclinical neurodevelopment in early childhood. Further research is necessary to elucidate the causal relationship and provide further evidence of a correlation between the ambient air pollution and neurobehavioral development to improve public health.
